# Aging increases metabolic capacity and reduces work efficiency during handgrip exercise in males

**DOI:** 10.1152/japplphysiol.00411.2022

**Published:** 2023-03-23

**Authors:** Anna Pedrinolla, Ole Kristian Berg, Tiril Tøien, Eivind Wang

**Affiliations:** ^1^Department of Neurosciences, Biomedicine and Movement Sciences, University of Verona, Verona, Italy; ^2^Department of Cellular, Computational and Integrative Biology—CIBIO, University of Trento, Trento, Italy; ^3^Faculty of Health and Social Sciences, Molde University College, Molde, Norway; ^4^Department of Physical Medicine and Rehabilitation, St. Olav’s University Hospital, Trondheim, Norway

**Keywords:** exercise, forearm, muscle diffusion, V̇o_2max_, V̇o_2peak_, vascular conductance, vascular function

## Abstract

Maximal oxygen uptake and exercise performance typically decline with age. However, there are indications of preserved vascular function and blood flow regulation during arm exercise. Yet, it is unknown if this potential physiological preservation with age is mirrored in peripheral metabolic capacity and V̇o_2_/W ratio. Thus, to investigate the effects of aging in the arms, we measured metabolic and vascular responses to 6-min bouts of dynamic handgrip exercise at 40% and 80% of maximal work rate (WR_max_) in 11 young (26 ± 2 yr) and 12 old (80 ± 6 yr) males, applying Doppler-ultrasound combined with blood samples from a deep forearm vein. At baseline, the old had a larger arterial diameter compared with young (*P* < 0.001). During exercise, the two groups reached the same WR_max_. V̇o_2_, blood flow, and oxygen supply were higher (40%WR_max_; 80%WR_max_, all *P* < 0.01), and arteriovenous oxygen difference was lower (80%WR_max_, *P* < 0.02), in old compared with young. Old also had a higher oxygen excess at 80%WR_max_ (*P* < 0.01) than young, whereas no difference in muscle diffusion or oxygen extraction was detected. Only young exhibited an increase in intensity-induced arterial dilation (*P* < 0.05), and they had a lower mean arterial pressure than old at 80%WR_max_ (*P* < 0.001). V̇o_2_/W (40%WR_max_; 80%WR_max_) was reduced in old compared with young (both *P* < 0.05). In conclusion, in old and young males with a similar handgrip WR_max_, old had a higher V̇o_2_ during 80%WR_max_ intensity, achieved by an increased blood flow. This may be a result of the available cardiac output reserve, compensating for reduced work efficiency and attenuated vascular response observed in old.

**NEW & NOTEWORTHY** Contrasting the typically observed decline in V̇o_2max_ with age, the current study reveals an age-related increase in forearm metabolic capacity during handgrip exercise in old, mediated by an increased forearm blood flow. Exercise with a small muscle mass in arms, where central components of the oxygen transport are not limiting, allows old to attain a similar maximal work rate as young despite their increased V̇o_2_/W ratio.

## Graphical abstract

**Figure:**
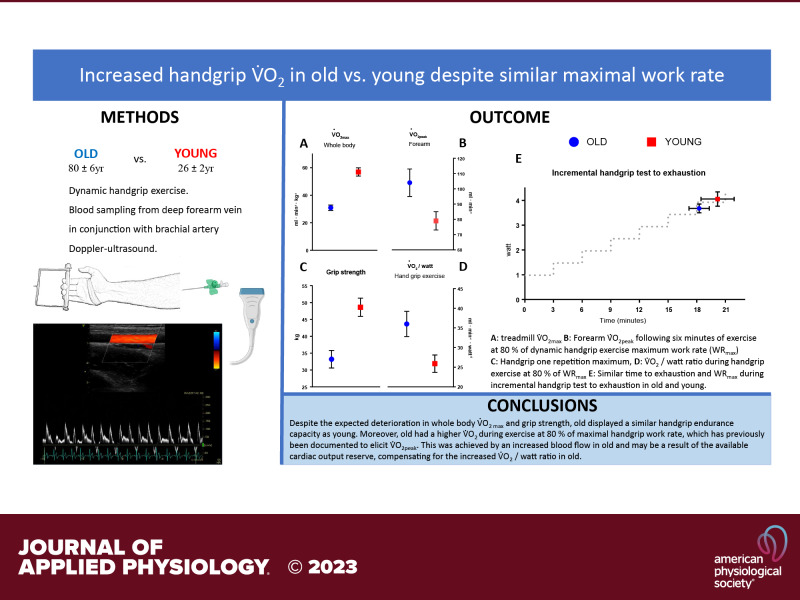

## INTRODUCTION

Maximal oxygen uptake (V̇o_2max_) typically declines with ∼1% per year after the third decade, and it is a key contributor to decreased exercise capacity (1). What determines V̇o_2max_ has been one of the most investigated research questions in physiology, with age-related reductions being attributed to both central and peripheral components of the oxygen transport during whole body exercise. The central limitations have mainly been explained by impaired maximal heart rate and stroke volume (2, 3), whereas vascular function and metabolic demand, along with morphological and structural alterations within skeletal muscle, have been forwarded as peripheral limiting factors (4–6).

As the size of exercising muscle mass decreases, the relative influence of peripheral factors becomes larger (7). In accordance with this notion, isolated small muscle mass exercise models, such as single-leg plantar flexion, knee extension, and handgrip flexion have been of great value to provide insight into peripheral age-related alterations in the exercising muscle bed (8–10). A highly interesting observation from studies applying these isolated small muscle mass models is that the arms appears to exhibit different signs of peripheral vascular and exercise capacity aging compared with the legs (11). In contrast to the quadriceps, old and young individuals are shown to have a similar forearm muscle mass, maximal work rate (WR_max_), and exhibit similar forearm blood flow and vascular conductance, in response to handgrip exercise (12, 13). However, it is unknown if these striking peripheral vascular and exercise capacity similarities in the forearms of young and old are mirrored in peak oxygen uptake (V̇o_2peak_) and muscle V̇o_2_/W ratio.

The indications that aging appears to not affect blood flow and macrovascular function in the forearm musculature warrants further investigation. In particular, since it is unknown if potentially preserved blood flow and vascular function may, in combination with peripheral metabolic function, result in a preserved peripheral V̇o_2peak_ and V̇o_2_/W ratio. Therefore, the aim of the current study was to determine the age-related metabolic and vascular responses to dynamic handgrip exercise of moderate intensity (40% WR_max_) to high intensity (80% WR_max_), where the latter has been documented to correspond to V̇o_2peak_ (14). Consequently, we tested the following hypotheses: compared with young individuals, old subjects would *1*) have no difference in forearm V̇o_2_ and *2*) exhibit no difference in muscle V̇o_2_/W.

## METHODS

### Subjects

Eleven young males and twelve old males (Table 1) volunteered to participate in the study after being informed of potential risks and discomfort and signing written informed consents. All participants had a dominant right arm and reported to be moderately trained and participate in regular physical activity two to three times a week, but no specific upper body strength training or specific endurance training of the forearms. Subjects were healthy and nonsmoking and included in the study when free of any disease or chronic conditions, free of any medications, and free of any conditions that would affect the ability to perform assessments and test procedures. The current study was approved by the Regional Committee for Medical and Health Research Ethics in Norway and was carried out in accordance with the Declaration of Helsinki.

**Table 1.** Subject characteristics

	Young	Old	
	*n* = 11	*n* = 12	*P* Value
Age, yr	26 ± 2	80 ± 6	**<0.001**
Weight, kg	82.3 ± 8.6	80.8 ± 12.1	0.712
Height, cm	179.9 ± 5.4	178.9 ± 7.4	0.293
Pulmonary V̇o_2max_, mL·min^−1^·kg^−1^	56.9 ± 9.8	30.9 ± 6.9	**<0.001**
tHb, g·dL^−1^	14.5 ± 0.9	13.6 ± 0.9	**0.048**
$Sp_{O_{2}}$, %	98.6 ± 0.1	97.1 ± 0.7	0.792
Resting arterial diameter, cm	0.45 ± 0.05	0.50 ± 0.02	**0.001**

Data are presented as means ± SD. $Sp_{O_{2}}$, oxygen saturation; tHb, total hemoglobin; V̇o_2max_, maximal oxygen consumption.

### Study Timeline

Participants reported to the laboratory on two separate occasions with a minimum of 24 h between sessions. Anthropometric data, treadmill V̇o_2max_, forearm one repetition maximum (1RM), and WR_max_ were assessed on the first day in the laboratory. On the second day, fixed load exercise tests of 40% and 80% of WR_max_ were conducted. The 80% WR_max_ intensity was chosen as it has previously been demonstrated to elicit forearm V̇o_2peak_ in this experimental setup, and as such the V̇o_2_ at the end of the 80% WR_max_ exercise bout is an indication of V̇o_2peak_ (14).

### Anthropometric Measurements

Body mass and height were measured using a digital weight scale and a manual height scale. Forearm volume was measured by fluid displacement, whereas forearm lean muscle mass was calculated based on measurements of mean skinfold thickness (*S*_mean_) and mean forearm circumferences (*O*_mean_) as previously described (15), using the following formula:

(*1*) $$\text{Forearm lean muscle mass}=[\text{Forearm volume}\cdot 0.871-[\left(S_{\text{mean}}-0.04\right)\cdot 2^{-1}]\cdot \text{length}\cdot O_{\text{mean}}]$$

### Maximal Oxygen Consumption

As an assessment of the participants’ general physical activity, whole body V̇o_2max_ was determined using a Metamax II gas analyzer (Cortex Biophysik, Leipzig, Germany). An incremental treadmill protocol was applied (Woodway PPS Med, Woodway USA, Waukesha, WI) starting with 5% inclination, and where speed and/or inclination was increased each minute until exhaustion. V̇o_2max_ was considered achieved when two or more of the following criteria were met: an asymptote in oxygen uptake development despite increases in workload, respiratory exchange ratio of 1.10, and/or being within 5 beats·min^−1^ of maximal heart rate if this was known (16).

### Handgrip Experimental Design and Testing

Dynamic handgrip data were obtained applying a custom-made handgrip device with the subjects placed in a supine position on a bench with the arm resting fully extended lateral to the body at the level of the heart. The handgrip device consisted of an ergonomic 2-cm-diameter grip bar attached with a wire to an apparatus with a weight stack with the ability to adjust the resistance with increments of 0.25 kg for dynamic work. The range of movement was 5 cm in both concentric and eccentric phase, for a total of 10 cm for a whole movement cycle.

Handgrip WR_max_ was determined as previously described (15, 17). Briefly, the starting workload was raising and lowering a 2.0 kg weight, to which 1.0 kg was added every third minute until exhaustion. Emphasis was made throughout the test to exclude recruitment of upper body muscles not relevant for the contraction movement.

After two warmup sets of 10 repetitions at a light to moderate weight, 1RM was determined for dynamic handgrip contraction and defined as the highest load with a single fully executed contraction as previously described (17). 1RM served as a measure to enable comparison of the subjects’ maximal strength with the loads that were achieved during the incremental WR_max_ test.

The second test day started with the insertion of a catheter in the antecubital vein, and the brachial artery was visualized and marked before exercise. Subsequently, subjects started with the standardized workloads of 40% and 80% of WR_max_, in a randomized order. The duration was 6 min for each workload, and the contraction frequency (0.5 Hz) was guided by a metronome. The two different workloads were separated by 60 min of complete rest.

### Peripheral Hemodynamics

Blood velocity and vessel internal diameter in the brachial artery were measured utilizing triplex mode Doppler-ultrasound (Vivid E9, GE Healthcare) with a linear 4–12 MHz probe (GE 11L), as previously described (15). Velocity was measured as a time average across the 16-s recordings obtained at *minute 5* and *6*. Vessel diameter was calculated from the B-mode recording based on six frames with good image quality from each recording (end of *minute 5* and *6*), three frames from the concentric contraction phase, and three from the eccentric phase. In each of the six frames the respective diameter was an average of six diameter measures. Diameter measurements from every frame were then averaged and used as brachial artery diameter for that 16-s recording. The color Doppler functionality expedited localization of the brachial artery and helped in detecting bifurcations, so that the sample volume could be placed above these. During exercise, especially in the relaxation phase coupled with systole, the magnitude of color Doppler representation masked the two-dimensional (2-D) B-mode representation of the vessel wall. Thus, to ensure a clear horizontal visualization of the border between the intima and the lumen of the vessel, duplex image recordings of 16-s duration were taken at baseline, and during the final minute of exercise, with corresponding venous blood samples, using the Vivid E9 system (GE healthcare). Muscle blood flow ($\dot{Q}$m) was calculated according to the following equation:

(*2*) $$\dot{Q}\text{m }=\text{ π}\cdot {\left(D_{\text{v}}\;\cdot {\text{ 2}}^{-1}\right)}^{2}\cdot V_{\text{b}}$$

where $\dot{Q}$m represents blood flow in a given vessel, *V*_b_ is the blood velocity in the vessel, and *D*_v_ is the vessel diameter at the point of velocity measurement. In addition, to give an estimate of the mechanical stress on the arterial wall, shear rate was calculated according to the following equation (18):

(*3*) $$\text{Shear rate }=\text{ 8}\cdot V_{\text{b}}/D_{\text{v}}$$

Arterial dilation was calculated as a percentage change of the diameter recorded at 5 and 6 min of exercise in relation to the baseline diameter (19). Arterial dilation was then normalized for shear rate.

### Blood Gas Measurements and Blood Lactate

An 18-G venous cannula (BD Venflon, Beckton Dickinson, Franklin Lakes, NJ) was inserted at the antecubital fossa into a deep forearm vein. Blood samples (1 mL) were taken in heparinized blood gas syringes (Portex Line Draw Plus, Smiths Medical, St. Paul, MN) at baseline, and at the 5th and 6th minute of exercise. The venous blood samples were gently mixed with the dry heparin in the syringe and preserved on ice. Blood samples were analyzed within 30 min after being drawn using an ABL800 Flex blood gas analyzer (Siemens Healthcare, Erlangen). Arteriovenous oxygen difference (a-vO_2diff_) was calculated from the difference between arterial ($Ca_{O_{2}}$) and venous ($Cv_{O_{2}}$) blood oxygen content, which were calculated as:

(*4*) $$\text{Blood oxygen content}=\left[1.39\left(\text{Hb}\right)\cdot \left({\text{Sp}}_{O2}/100\right)+0.003\cdot {\text{Po}}_{2}\right]$$

Oxygen saturation (${\text{Sp}}_{\text{O2}}$) was measured by a finger-pulse oximeter on the nonexercising arm. Mean capillary Po_2_ and muscle oxygen diffusing capacity at in the final minute at 80% WR_max_ was calculated as previously described by Bohr integration and Fick’s law of diffusion, with the assumption of eliciting V̇o_2peak_ (20, 21).

Consequently, forearm oxygen uptake (V̇o_2_) was estimated from recorded $\dot{Q}$m in the brachial artery and a-vO_2diff_, according to the formula:

(*5*) $${\dot{V}O}_{2}=\dot{Q}m\cdot {\text{a}‐\text{vO}}_{2\text{diff}}$$

Oxygen supply to forearm muscle was estimated as the product of $Ca_{O_{2}}$ and $\dot{Q}$m, whereas oxygen excess was calculated as the product of $Cv_{O_{2}}$ and $\dot{Q}$m (22). Oxygen extraction expresses the ratio between oxygen uptake and arterial oxygen supply and was calculated by dividing a-vO_2diff_ by the arterial oxygen concentration and expressed as a percentage.

### Muscle V̇o_2_/W and Work Efficiency

Both the submaximal V̇o_2_/W ratio at 40% WR_max_ and the V̇o_2_/W ratio at 80% of WR_max_ were calculated by dividing the V̇o_2_ with the corresponding external work in watt. In addition, the work efficiency defined as the ratio of external work to the corresponding internal energy expenditure was calculated for the submaximal 40% WR_max_ intensity. External work was given by the exercise load in kilograms, the range of motion, and the frequency. The energy expenditure was given as the average forearm V̇o_2_ the last minute of the 6-min work period. In accordance with previous literature, external work and forearm energy expenditure of the submaximal work rate were converted to kilocalories using a fixed conversion rate before the calculation of work efficiency and expressed as a percentage (17).

### Central Hemodynamics

Mean arterial blood pressure (MAP) and heart rate were measured beat by beat by photoplethysmography on the fingers (PortaPres, Finapres Medical Systems, Amsterdam, The Netherlands) on the nonexercising arm. It was placed in a relaxed position on the side of the subject at the level of the heart throughout the test while the heart rate was measured with three-lead electrocardiography (ECG). MAP for each of the handgrip exercise workloads were calculated as averages over the final minute of exercise corresponding to the Doppler-ultrasound recordings. Vascular conductance was calculated as $\dot{Q}$m/MAP (14).

### Statistical Analysis

All statistical analyses were performed with Sigma PLOT Windows Version 14.0 (Systat Software, Chicago, IL). Data are presented as means ± SD, if not stated otherwise. First, normality was assessed by the Shapiro–Wilk test. A one-way (1 × 2) analysis of variance (ANOVA) was applied to baseline characteristics to detect between-groups differences. A two-way (2 × 2) ANOVA, with “Group” (old and young) as within-group factor, and “Intensity” (40% WR_max_ and 80% WR_max_) as between-group factors was applied to all variables. A three-way (2 × 2 × 7) ANOVA with “Group” (old and young), “Intensity” (40% and 80%), and “Time” (Baseline, 1′, 2′, 3′, 4′, 5′, 6′) was applied to mean arterial pressure and heart rate. In the presence of significant effects, a multiple-comparisons test with Holm–Sidak’s correction was performed. The familywise α level for significance was set at 0.05 (two-tails), with Holm–Sidak’s correction when needed, for all the analyses.

## RESULTS

Young had a higher whole body V̇o_2max_ than old [Diff of means (*D*) = 26.027, *t* = 6.527, *P* < 0.001]. Between-groups differences were found for total hemoglobin (tHb), with old showing lower values compared with young (*D* = 0.955, *t* = 2.013, *P* = 0.048); and for resting brachial artery diameter, where old exhibited a larger resting diameter compared with young (*D* = 0.059, *t* = 3.373, *P* = 0.001). ${\text{Sp}}_{\text{O2}}$ was not different between young and old at baseline, and it remained stable across all intensities in both groups (Table 1). Supplemental Material (see https://doi.org/10.6084/m9.figshare.22289149) shows individual values of main variables and main effects.

### Handgrip Maximal Work Rate

No differences were detected between young and old in WR_max_ (Table 2). The 40% WR_max_ workload was equivalent to 3.3 ± 0.8 in young and 3.1 ± 0.5 kg in old (1.6 ± 0.4 and 1.5 ± 0.2 W), and 80% WR_max_ was equivalent to 6.6 ± 1.6 in young and 6.2 ± 1.0 kg in old (3.2 ± 0.7 and 3.0 ± 0.5 W). As for the WR_max_, 40% WR_max_ and 80% WR_max_ were not different between groups. Old exhibited a lower 1RM compared with young (*D* = 15.389, *t* = 3.952, *P* < 0.001), whereas there was no difference between the groups in forearm muscle volume or mass (Table 2).

**Table 2.** Handgrip WR_max_ and maximal strength

	Young	Old	
	(*n* = 11)	(*n* = 12)	*P* Value
WR_max_, W	4.0 ± 0.9	3.7 ± 0.6	0.228
Forearm lean muscle mass, kg	0.9 ± 0.1	1.1 ± 0.2	0.470
Handgrip 1RM, kg	48.6 ± 8.9	33.2 ± 8.9	**<0.001**

Data are presented as means ± SD. 1RM, dynamic one repetition handgrip maximal strength; WR_max_, maximal work rate.

### Forearm Oxygen Uptake

**Figure 1.**
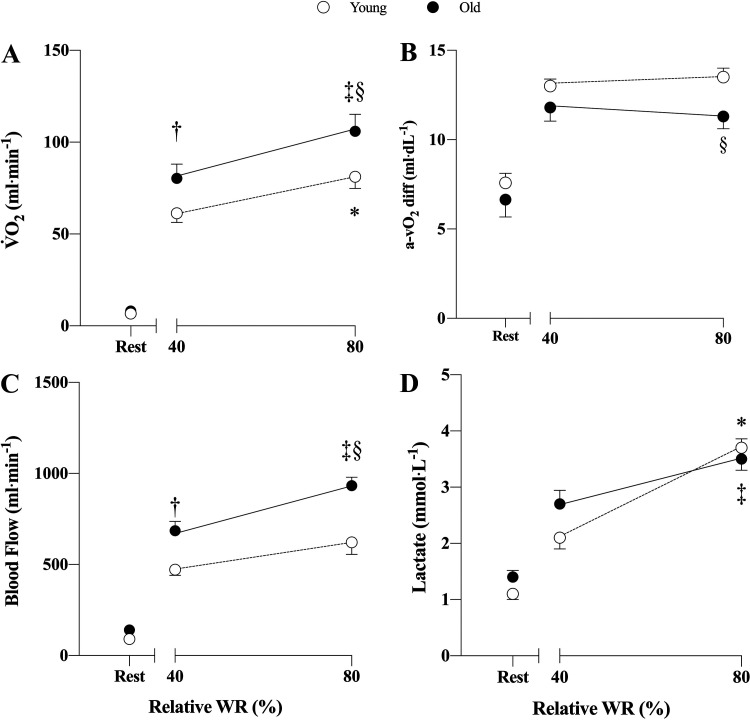
Forearm oxygen uptake (V̇o_2_, *A*), arterial-venous oxygen difference (a-vO_2_diff, *B*), brachial artery blood flow (*C*), and lactate concentration (*D*) in young (*n* = 11) and old (*n* = 12) males. Values are represented as means ± SE. *P < 0.05 in young group 40% vs. 80%. ‡*P* < 0.05 in old group 40% vs. 80%. †*P* < 0.05 at 40% young vs. old. §*P* < 0.05 at 80% young vs. old. Main effect for “group,” *P* < 0.001 in *A*, *C*, and *D*, for “intensity,” *P* < 0.001 in *A*, *C* and *D*, for “group × intensity,” *P* = 0.005 in *B*. WR, work rate.

V̇o_2_ increased from baseline to 40% WR_max_ and 80% WR_max_ in both young (*D* = 53.220, *t* = 6.355, *P* < 0.001; *D* = 72.387, *t* = 8.644, *P* < 0.001, respectively) and old (*D* = 72.944, *t* = 9.098, *P* < 0.001; *D* = 96.176, *t* = 11.995, *P* < 0.001, respectively). Differences were detected in both young and old at 80% WR_max_ compared with 40% WR_max_ (*D* = 19.167, *t* = 2.289, *P* = 0.025; *D* = 23.232, *t* = 2.897, *P* = 0.005, respectively). Also, between-group differences for old and young were found at both 40% WR_max_ and 80% WR_max_ (*D* = 21.453, *t* = 2.617, *P* = 0.011; *D* = 25.517, *t* = 3.113, *P* = 0.003, 40% and 80%, respectively), with old exhibiting higher V̇o_2_ values than young at both intensities (Fig. 1*A*). No difference was found when comparing V̇o_2_ at *minute 5* to *minute 6* in neither young nor old.

### Forearm Arteriovenous Oxygen Difference

Arteriovenous oxygen difference increased from baseline to 40% WR_max_ and 80% WR_max_ in both young and old (*D* = 5.325, *t* = 6.247, *P* < 0.001; *D* = 5.827, *t* = 6.836, *P* < 0.001, respectively). Differences between the two work intensities were not detected in any group. Between-group differences were observed only at 80% WR_max_ (*D* = 2.015, *t* = 2.415, *P* = 0.019), with young showing higher a-vO_2diff_ compared with old (Fig. 1*B*).

### Forearm Blood Flow

Forearm blood flow increased from baseline to 40% WR_max_ and 80% WR_max_ in both young (*D* = 381.705, *t* = 6.366, *P* < 0.001; *D* = 530.895, *t* = 8.855, *P* < 0.001, respectively) and old (*D* = 545.645, *t* = 9.505, *P* < 0.001; *D* = 792.289, *t* = 13.802, *P* < 0.001, respectively). Differences were detected in both groups at 80% WR_max_ compared with 40% WR_max_ (*D* = 149.190, *t* = 2.488, *P* = 0.015; *D* = 246.644, *t* = 4.297, *P* < 0.001 in young and old, respectively). Also, differences between old and young were observed at both intensities (*D* = 213.895, *t* = 3.644, *P* < 0.001; *D* = 311.340, *t* = 5.305, *P* < 0.001, 40% and 80%, respectively), with old having higher forearm blood flow than young (Fig. 1*C*).

### Lactate Concentration in Blood

Lactate concentration significantly increased from rest to 40% and 80% in both young (*D* = 0.982, *t* = 2.658, *P* = 0.010, and *D* = 2.582, *t* = 6.990, *P* < 0.001, respectively) and old (*D* = 1.250, *t* = 3.535, *P* = 0.002, and *D* = 2.125, *t* = 6.009, *P* < 0.001, respectively). Both groups exhibited significant difference at 80% compared with 40% (*D* = 1.600, *t* = 4.332, *P* < 0.001 and *D* = 0.875, *t* = 2.474, *P* = 0.016 in young and old, respectively) but no between-group difference was detected at 40% or 80% (Fig. 1*D*).

### Muscle V̇o_2_/W and Work Efficiency

**Figure 2.**
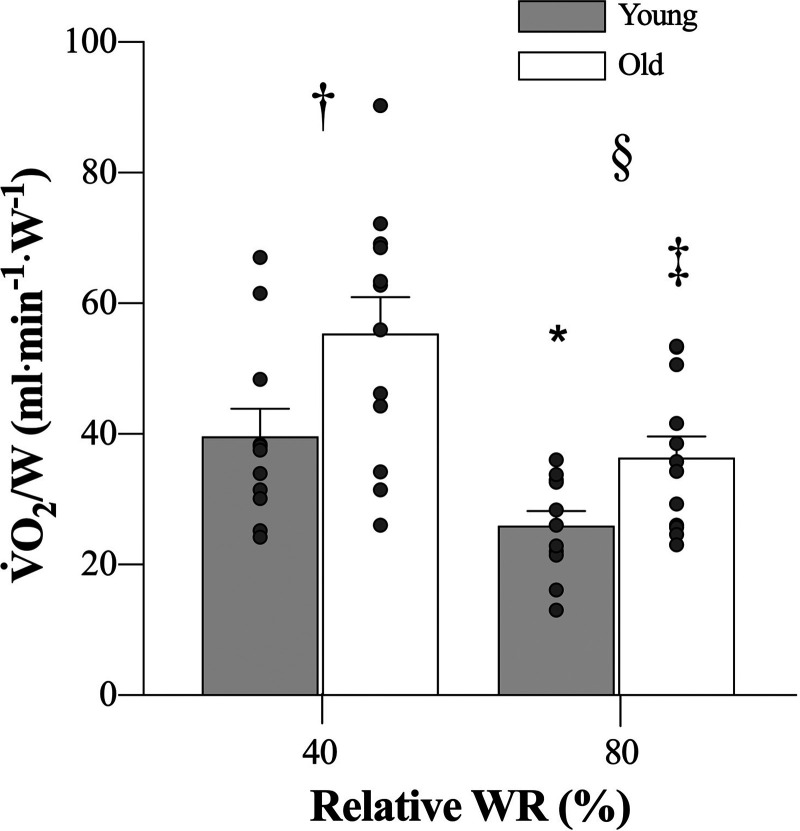
Oxygen consumption to watts (V̇o_2_/W) ratio in young (*n* = 11) and old (*n* = 12) males at 40% and 80% of handgrip peak work rate. Main effect for “group,” *P* < 0.001 and “intensity,” *P* < 0.001. Values are represented as means ± SD. *P < 0.05 in young group 40% vs. 80%. ‡*P* < 0.05 in old group 40% vs. 80%. †*P* < 0.05 at 40% young vs. old. §*P* < 0.05 at 80% young vs. old.

The ratio of muscle V̇o_2_ to watt for young and old was lower in both groups at 80% WR_max_ versus 40% WR_max_ (*D* = 28.471, *t* = 2.534, *P* = 0.015 in young and *D* = 40.051, *t* = 3.737, *P* < 0.001 in old). At both intensities, old displayed a higher V̇o_2_/W ratio compared with young (40% WR_max_: *D* = 34.183, *t* = 2.469, *P* = 0.022; 80% WR_max_: *D* = 7.841, *t* = 2.770, *P* = 0.006, Fig. 2). The difference in work efficiency for old compared with young was 2.8 ± 1 versus 4.2 ± 1.2%, respectively (*D* = 1.410, *t* = 2.58, *P* = 0.012).

### Arterial Dilation, Shear Rate, and Arterial Dilation Normalized for Shear Rate

Only young exhibited a difference in arterial dilation between 40% WR_max_ and 80% WR_max_ (*D* = 2.995, *t* = 2.078, *P* = 0.042), whereas no differences were detected in old. Young displayed a tendency (*P* = 0.055) of higher arterial dilation than old at 80% WR_max_ (Table 3).

**Table 3.** Arterial diameter, arterial dilation, shear rate, mean arterial pressure, and vascular conductance during 40% and 80% WR_max_

	Young		Old	
	(*n* = 11)		(*n* = 12)	
	40%	80%	40%	80%
Arterial diameter, cm	0.48 ± 0.05	0.49 ± 0.07	0.53 ± 0.03†	0.55 ± 0.02§
Arterial dilation, %	6.9 ± 3.7	9.2 ± 5.3*	5.7 ± 3.6	6.4 ± 3.2
Shear rate, s^−1^	724.0 ± 52.5	877.4 ± 66.9*	781.4 ± 66.3	976.0 ± 62.1‡
MAP, mmHg	100.3 ± 3.9	104.7 ± 10.3	107.4 ± 5.5	127.9 ± 5.0‡§
Vascular conductance, mL·mmHg^−1^·min^−1^	4.8 ± 0.4	7.2 ± 0.5	6.6 ± 0.6	7.3 ± 0.4

Data are presented as means ± SD. MAP, mean arterial pressure; WR_max_, maximal work rate.

*P < 0.05 in young group 40% vs. 80%; ‡*P* < 0.05 in old group 40% vs. 80%; †*P* < 0.05 at 40% young vs. old; §*P* < 0.05 at 80% young vs. old.

No between-group differences were found in shear rate. However, both young and old exhibited differences between 40% WR_max_ and 80% WR_max_ (*D* =153.336, *t* = 2.003, *P* = 0.046; *D* = 194.56, *t* = 2.694, *P* = 0.009, respectively; Table 3).

No between- or within-group differences were detected when arterial dilation was normalized for shear rate.

### Oxygen Supply, Extraction, and Excess

**Figure 3.**
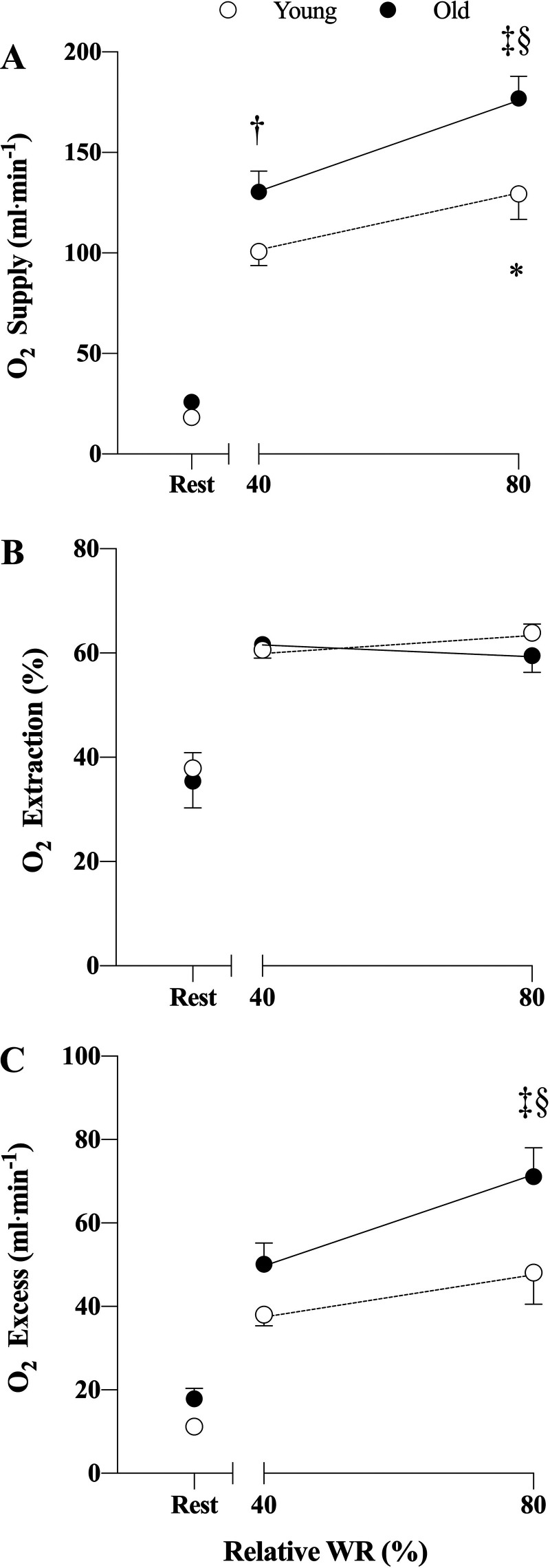
Oxygen (O_2_) supply (*A*), extraction (*B*), and excess (*C*) in young (*n* = 11) and old (*n* = 12) males. Values are represented as means ± SE. *P < 0.05 in young group 40% vs. 80%. ‡*P* < 0.05 in old group 40% vs. 80%. †*P* < 0.05 at 40% young vs. old. §*P* < 0.05 at 80% young vs. old. Main effect for “group,” *P* < 0.001 in *A* and *P* = 0.002 in *C*, for “intensity,” *P* < 0.001 in *A*–*C*. WR, work rate.

Mirroring V̇o_2_ and blood flow, oxygen supply also increased from rest to both 40% WR_max_ and 80% WR_max_ in young (*D* = 81.282, *t* = 6.633, *P* < 0.001, and *D* = 109.100, *t* = 8.903, *P* < 0.001) and old (*D* = 105.136, *t* = 8.961, *P* < 0.001, and *D* = 149.371, *t* = 12.732, *P* < 0.001). An increase from 40% WR_max_ to 80% WR_max_ was detected in young (*D* = 27.818, *t* = 2.270, *P* = 0.027) and old (*D* = 44.235, *t* = 3.77, *P* < 0.001). Between-group differences were observed at both 40% WR_max_ and 80% WR_max_, with old exhibiting higher oxygen supply compared with young at both intensities (*D* = 31.917, *t* = 2.661, *P* = 0.010; and *D* = 48.334, *t* = 4.029, *P* < 0.001 at 40% and 80%, respectively; Fig. 3*A*).

Oxygen extraction was higher at 40% WR_max_ and 80% WR_max_ compared with rest in both young (*D* = 0.229, *t* = 5.917, *P* < 0.001; and *D* = 0.262, *t* = 6.762, *P* < 0.001) and old (*D* = 0.263, *t* = 7.104, *P* < 0.001; and *D* = 0.238, *t* = 6.439, *P* < 0.001). However, no differences were detected between the two intensities within groups or between groups (Fig. 3*B*).

O_2_ excess was different at 40% WR_max_ and 80% WR_max_ compared with rest in both young (*D* = 26.827, *t* = 3.358, *P* = 0.003; and *D* = 36.928, *t* = 4.623, *P* < 0.001) and old (*D* = 32.196, *t* = 4.654, *P* < 0.001; and *D* = 53.201, *t* = 7.690, *P* < 0.001). Oxygen excess was higher at 80% WR_max_ compared with 40% WR_max_ in old (*D* = 21.005, *t* = 3.036, *P* = 0.004), but this was not evident in young. Between-group difference was detected at 80% WR_max_ with old showing higher values than young (*D* = 22.912, *t* = 3.006, *P* = 0.003), but not at 40% WR_max_ (Fig. 3*C*).

There was no difference between old and young in muscle oxygen diffusing capacity. Old displayed a higher mean capillary Po_2_ compared with young (*P* = 0.033), whereas there was no difference in venous Po_2_ between the groups at the end of the 80% WR_max_ exercise (3.54 ± 0.29 vs. 3.74 ± 0.60 kPa, for young and old, respectively).

### Mean Arterial Pressure and Vascular Conductance

MAP did not increase from rest to 40% WR_max_ or 80% WR_max_ in young, whereas old exhibited an increase in MAP from rest to 80% WR_max_ (*D* = 33.865, *t* = 4.235, *P* < 0.001) and from 40% WR_max_ to 80% WR_max_ (*D* = 20.530, *t* = 2.567, *P* = 0.026). No between-group differences were detected at 40% WR_max_, but at 80% WR_max_ old had a higher MAP than young (*D* = 23.252, *t* = 2.759, *P* = 0.008, Table 3). Time effect was found at both intensities until the third minute in young (*F* = 3.975, *P* < 0.001), and until the fourth minute in old (*F* = 4.5887, *P* < 0.001) after which no significant difference in MAP was observed for the following timepoints MAP at both intensities. Time effect was also found for HR which increased in both groups until the third minute of exercise (*F* = 25.876, *P* < 0.001) after which no significant difference in HR was observed for the following timepoints at both intensities.

Vascular conductance increased from rest to 40% WR_max_ and 80% WR_max_ in both young (*D* = 3.796, *t* = 3.221, *P* < 0.004; *D* = 6.169, *t* = 4.966, *P* < 0.001, respectively) and old (*D* = 4.992, *t* = 4.239, *P* < 0.001; *D* = 5.775, *t* = 4.900, *P* < 0.001, respectively). No within- or between-group differences were detected at the two intensities.

## DISCUSSION

The effects of aging in humans have been indicated to be limb-specific. In contrast to the legs, there are indications of preserved muscle mass in the arms, along with preserved exercise capacity, vascular function, and blood flow. To provide new insight into the role of peripheral components in age-related oxygen transport in arms, we investigated if signs of absence of aging in arms included preserved metabolic capacity and muscle V̇o_2_/W ratio by applying handgrip exercise at moderate and high intensity. In accordance with previous studies, old and young in the current study had a similar forearm muscle mass and handgrip WR_max_, and the main findings were that *1*) aging increased forearm V̇o_2_; *2*) the increased V̇o_2_ was mediated by a higher maximal blood flow to exercising musculature; and *3*) the muscle V̇o_2_/W ratio was increased, and maximal muscle strength reduced, in old compared with young. In combination, these results reveal an arm-specific age-related higher metabolic capacity achieved through an increased maximal blood flow. This peculiarity may be compensating for the age-related increase in the muscle V̇o_2_/W ratio, preserving the old’s ability to perform maximal work with a small muscle mass.

### Forearm Metabolic Capacity and Aging

To the authors’ knowledge, this is the first study to compare forearm V̇o_2_ during dynamic handgrip exercise in young and old. Surprisingly, handgrip exercise at the end of the 80% WR_max_ intensity, previously documented to correspond to handgrip V̇o_2peak_ (14), was higher in old compared with young. This finding challenges the putative assumption that V̇o_2peak_ declines with advancing age and contrasts observations in legs (8) or during whole body exercise (1). Indeed, a typical age-related reduction in whole body V̇o_2max_ of ∼1% per year was also observed between the groups in the present study. The handgrip V̇o_2_ values at 80% WR_max_ observed in young (81 ± 21 mL·min^−1^) with 0.9 kg of muscle mass correspond well with previous reports (14) from our laboratory, and is in accordance with what should be expected during a workload of 3.2 W (Fig. 2 in Ref. 15). Our results are also in accordance with the muscle mass normalized ∼79 mL·min^−1^·kg^−1^ V̇o_2peak_ that others have reported during arm exercise [gleaned from Fig. 4 in Calbet et al. (23)], strengthening the confidence with which we can assume that the measurements in the current study were correct and representative. The V̇o_2_ at 80% WR_max_ of 106 ± 32 mL·min^−1^ in the old was ∼30% higher in relation to the corresponding workload than what should be expected from previous data in young (15). Since old achieved a similar WR_max_ as young, it implies that old had a higher muscle V̇o_2_/W. This is in contrast to knee extension exercise, where a similar V̇o_2_/W relationship has been observed in old and young (8). The age-related increased muscle V̇o_2_/W relationship observed during high-intensity handgrip exercise in the present study was also observed during submaximal intensity (40% WR_max_), revealing a systematic higher metabolic cost of work from moderate to high-intensity exercise. This may explain why old had a similar WR_max_ as young, despite having a higher V̇o_2_ in the current study.

### Work Efficiency, Exercise Capacity, Muscle Mass, and Muscle Strength

Forearm muscle mass was similar between old and young in the current study. However, despite the similar muscle mass, the maximal strength in the old was only 68% that of the young. The latter observation corresponds closely with previous normative age-related values of handgrip strength from large populational studies (24, 25), and maybe an explanation for the attenuated muscle V̇o_2_/W relationship observed in old. Indeed, a recent study from our group demonstrated that maximal muscle strength influences work efficiency during dynamic handgrip exercise (17). In line with this notion, such a relationship is in agreement with observations that relative strength of the arms correlates with gross mechanical efficiency during arm cycling (26), and also with what has been documented in the legs, where increased leg muscle strength in old led to a restoration of their impaired walking work efficiency (27).

As observed in the present study, the age-related loss of muscle strength is typically larger than the loss of muscle mass (28). First, this may be because neural factors play an important role in the age-related muscle strength deterioration (29). Second, it could also be due to alterations in the muscle fiber distribution with an age-related shift from type II to weaker, but more oxidative type I muscle fibers (27). Muscle fibers in old may also produce less force compared with young, even when normalized to fiber size (30). Considering these degenerative structural changes of muscle fibers associated with aging, along with potentially increased portion of type I fibers (27, 31), old may need to recruit a higher amount of muscle fibers to overcome external workloads compared with young. Third, previous studies have demonstrated that old exhibit an elevated ATP cost of contraction compared with activity-matched young both in plantar flexor and knee-extension exercise (32, 33). In combination, all these potential age-related alterations may reduce muscle strength and lead to an increased forearm V̇o_2_/W ratio and reduced work efficiency. Of notice, such alterations would counteract the improved work efficiency associated with work reliant on efficient oxidative type I fibers.

Interestingly, when limited more by central factors, as when performing whole body exercise, this age-associated difference would result in reduced exercise capacity. However, this appeared to not be the case when exercising with a small muscle mass in the arms, as both old and young reached a similar WR_max_. Although the muscles of old are weaker than young, previous studies have demonstrated that they fatigue relatively less than young in response to exercise (34). This may, again, be related to the mentioned shift toward a more enduring, yet slow, muscle phenotype. Taken together our results indicate a compensatory adaptation in the muscle bed of the old allowing them to match the work done by the stronger forearm of the young during the incremental handgrip test to exhaustion. This may indicate that age-related changes in the musculature could, during specific circumstances such as small muscle mass exercise, facilitate a higher reliance on, and capacity for, aerobic work, as previously suggested by Ferri et al. (35).

### Forearm Blood Flow and Oxygen Extraction

The higher V̇o_2_ for a given exercise intensity in older individuals was achieved through a higher blood flow. This finding is in contrast to the typical reduction observed in the legs (8). However, previous studies investigating handgrip exercise have revealed that blood flow during exercise (13), or in postcontraction hyperemia (12), is not reduced with aging. In fact, Donato et al. (13) reported a tendency for old to display a higher blood flow relative to forearm muscle mass compared with the young during the absolute handgrip work rates of 3 and 6 kg, similar to the 40% and 80% of WR_max_ in the current investigation. Importantly, the present study applied dynamic handgrip exercise, where the muscle is constantly under tension. The concentric and eccentric duty cycle elicits a higher metabolic demand than isometric exercise at similar torque (36), which may explain the high metabolic demand and forearm blood flow in the present study. In turn, the higher blood flow in old likely caused the increased oxygen excess and lower a-vO_2diff_ compared with young. Of notice, the higher forearm blood flow in old yielded a higher oxygen supply at both moderate and high exercise intensities, also when taking their lower [Hb] into account. The high forearm peak perfusion was enabled by an increased MAP in old, with the result being a similar forearm vascular conductance as observed in young. An unaltered vascular conductance with age during intensive handgrip exercise is in line with previous reports (13).

Assuming that ∼53% of the forearm is involved in handgrip exercise (37), the highest blood flow achieved during 80% WR_max_ was 160 mL^−1^·min·100 g^−1^ and 122 mL^−1^·min·100 g^−1^ in old and young, respectively, in the current study. These values are, using an estimated muscle mass of ∼4 kg for arms (38), in line with values of ∼130 mL^−1^·min·100 g^−1^ observed during double poling arm exercise in young cross-country skiers (39). Albeit, such a comparison should be made with caution since estimated active muscle mass is somewhat uncertain for the different arm exercise modalities. Yet, maximal blood flow in the forearm and the arms is considerably lower than the maximal values of 385 mL^−1^·min·100 g^−1^ previously observed during knee extension exercise in young athletes (40). Comparing maximal blood flow in young in the current study with previous studies, applying other exercise modalities, strengthens the confidence that the measurements of relatively high maximal blood flow during handgrip exercise in old are correct. The large difference between handgrip and quadriceps exercise may, in turn, be explained by the substantial hindrance of blood flow during handgrip muscle contractions (14).

Interestingly, the higher age-related forearm blood flow in the present study did not reduce muscle oxygen diffusive capacity, where factors such as inappropriate blood flow distribution, heterogeneity, mixing of blood sampled from active and inactive regions, or/and reduced mean transit time (41, 42) could potentially have blunted an already poor oxygen diffusion process in the arms. One possible explanation may be that the diffusion process was maintained because of a higher driving force (Po_2_) across the length of the available capillaries (43). Indeed, one previous study demonstrated an augmentation of muscle diffusive capacity in old when capillary blood flow, and thus O_2_ supply, to the active muscle bed was increased following ischemic plantar flexion (44).

### Vascular Responsiveness and Age

The increased forearm blood flow in the current study was an adumbration of preserved vascular function with advancing age. However, details of arterial dilation in old and young were not in support of this notion. Although old displayed the same arterial dilation in response to moderate and high intensity handgrip exercise, young exhibited an increased response to the latter. In addition, attenuated vascular responsiveness in old was also apparent as a clear tendency (*P* = 0.055) for young to have a higher dilation compared with the old at 80% of WR_max_. Interestingly, gleaning from Figs. 3 and 4 in Wray et al. (45), brachial artery flow-mediated dilation (FMD) in old seem to reach a plateau at ∼5% dilation, even after ischemic exercise superimposed on cuff occlusion. In that study, peak brachial artery shear rate was ∼330 s^−1^. A similar value of 217 s^−1^ during FMD in old following 4 wk of handgrip training was also reported in another study and resulted in a peak dilation of 4.2% (46). In the present study, during a dynamic handgrip exercise, we observed two- to threefold higher shear rates for old than reported in the previous studies, with 742 (40% WR_max_) and 877 s^−1^ (80% WR_max_). Although qualitatively different, a comparison of the present study with previous studies applying reactive hyperemia following cuff occlusion is interesting because all result in a dilation between ∼4% and 5% for old. Albeit a higher dilation has been reported in 10 yr younger individuals than in the present study, paralleled by a smaller baseline arterial diameter (10). Contrary to these observations, young are shown to exhibit a greater dilation in the range of ∼9%–10% following both cuff occlusion and handgrip exercise (14, 15, 47). A potential age-related ceiling for shear-induced vasodilation may be due to both structural changes of the conduit artery diameter as well as impaired bioavailability of vasoactive agents, such as nitric oxide. Thus, the increased MAP observed in old may potentially be a compensatory mechanism, for the diminished vascular responsiveness, enabling blood flow to meet the metabolic demand in the exercising forearm.

### Experimental Considerations

Although we did not verify that V̇o_2peak_ was achieved using a separate test in the current study, previous studies have documented that V̇o_2_ after 6 min of dynamic handgrip exercise at 80% of WR_max_ correspond to V̇o_2peak_. In the previous studies V̇o_2peak_ was achieved at task failure during exercise at 100% WR_max_ (14) or following an incremental handgrip exercise test to failure (15). Notably, in support of previous observations, individuals from both groups in the current study similarly exhibited signs of struggle to maintain contraction frequency, and not utilize upper body musculature to assist the exercise, toward the end of the 80% WR_max_ work rate. Furthermore, no difference in V̇o_2_ was observed from *minute 5* to *6*. Consistent with our previous observations, a recent study by Fenuta et al. (48) utilizing isometric handgrip contractions, which seem to produce lower absolute values of forearm V̇o_2_ and a somewhat longer incremental test than in our setup, demonstrated that V̇o_2_ was not different at 88.6 ± 8.2% versus 100% of WR_max_ during an incremental handgrip test to exhaustion. A supramaximal exercise test to exhaustion should however be considered in future studies to verify the achievement of forearm V̇o_2peak_ (49). In particular, comparison with peak values determined from models derived from studies on young subjects should be considered in light of potentially slowed V̇o_2_ kinetics in older individuals. Moreover, as blood flow was measured in the brachial artery, branching and maldistribution of blood flow in the old could have accounted for the increased blood flow. Sampling from a single forearm vein, although a well-established (41, 50) and reliable (15) method may not be representative of the total venous outflow. Alternatively, applying a methodology that measures locally in the exercising musculature, such as near-infrared spectroscopy (35) or ^31^P magnetic resonance spectroscopy (51), could be utilized to verify the measurements and investigate anaerobic components related to the exercise performance. Thus, our findings should be interpreted cautiously, and further studies are needed to verify the interesting indications of increased metabolic capacity in the forearm musculature of old during handgrip exercise. Moreover, this study included only men, and thus investigation in females is needed to shed light on potential sex differences in the aging of the forearm musculature. Finally, several of the comparisons in the discussion are made to studies conducted in the lower extremities. The results from these studies need to be corroborated in the upper extremities. Although the current investigation is one step in that direction, several other aspects of the response to exercise with the upper extremities with advancing age remain to be investigated. Particularly, further examination of whether different observations in the forearm and the lower extremities are truly limb-specific is warranted. Future investigations of lower extremities small muscle mass exercise, compared with handgrip exercise, e.g., plantar flexion, could be carried out on old and young subjects that are matched for muscle mass and work capacity.

### Conclusion

This study, in old and young males, revealed that aging results in increased forearm blood flow during handgrip exercise, and in turn higher V̇o_2_ values, even when approaching peak exercise capacity. This unique adaptation in arms appears to compensate for an age-related increase in V̇o_2_W ratio and decreased vascular responsiveness. Thus, enabling old to reach a similar WR_max_ as young.

## DATA AVAILABILITY

Data will be made available upon reasonable request.

## SUPPLEMENTAL DATA

10.6084/m9.figshare.22289149 Supplemental Material: https://doi.org/10.6084/m9.figshare.22289149.

## DISCLOSURES

No conflicts of interest, financial or otherwise, are declared by the authors.

## AUTHOR CONTRIBUTIONS

O.K.B. and E.W. conceived and designed research; A.P., O.K.B., T.T., and E.W. performed experiments; A.P., O.K.B., and E.W. analyzed data; A.P., O.K.B., T.T., and E.W. interpreted results of experiments; A.P., O.K.B., and E.W. prepared figures; A.P., O.K.B., T.T., and E.W. drafted manuscript; A.P., O.K.B., T.T., and E.W. edited and revised manuscript; A.P., O.K.B., T.T., and E.W. approved final version of manuscript.
